# Oleanolic Acid Alleviates Cerebral Ischemia/Reperfusion Injury via Regulation of the GSK-3β/HO-1 Signaling Pathway

**DOI:** 10.3390/ph15010001

**Published:** 2021-12-21

**Authors:** Kaili Lin, Zhang Zhang, Zhu Zhang, Peili Zhu, Xiaoli Jiang, Ying Wang, Qiudi Deng, Ken Kin Lam Yung, Shiqing Zhang

**Affiliations:** 1 School of Public Health, Guangzhou Medical University, Guangzhou 511436, China; lin_kaili@gzhmu.edu.cn; 2 Department of Biology, Faculty of Science, Hong Kong Baptist University (HKBU), Kowloon Tong, Hong Kong 999077, China; 17423015@life.hkbu.edu.hk (Z.Z.); 15485412@life.hkbu.edu.hk (Z.Z.); pellyzhu@hkbu.edu.hk (P.Z.); 20482108@life.hkbu.edu.hk (X.J.); 18482937@life.hkbu.edu.hk (Y.W.); 3 Golden Meditech Center for Neuro Regeneration Sciences, HKBU, Kowloon Tong, Hong Kong 999077, China; 4 Department of Biochemistry and Molecular Biology, GMU-GIBH Joint School of Life Sciences, Guangzhou Medical University, Guangzhou 511436, China; 5 JNU-HKUST Joint Laboratory for Neuroscience and Innovative Drug Research, College of Pharmacy, Jinan University, Guangzhou 510632, China

**Keywords:** oleanolic acid, cerebral ischemia/reperfusion, antioxidant properties, GSK-3β/HO-1 pathway

## Abstract

Oleanolic acid (OA), a bioactive ingredient of *Panax ginseng*, exhibits neuroprotective pharmacological effects. However, the protective role of OA in cerebral ischemia and involved mechanisms remain unclear. This study attempted to explore the therapeutic effects of OA both in vitro and in vivo. OA attenuated cytotoxicity and overproduction of intracellular reactive oxygen species (ROS) by regulation of glycogen synthase kinase-3β (GSK-3β)/heme oxygenase-1 (HO-1) signal in oxygen-glucose deprivation/reoxygenation (OGD/R)-exposed SH-SY5Y cells. Additionally, OA administration significantly reduced the area of cerebral infarction and the neurological scores in the rat models of cerebral ischemia with middle cerebral artery occlusion (MCAO). The OA administration group showed a higher percentage of Nissl^+^ and NeuN^+^ cells, along with lower TUNEL^+^ ratios in the infarct area of MCAO rats. Moreover, OA administration reduced ROS production while it suppressed the GSK-3β activation and upregulated the HO-1 expression in infarcted tissue. Our results illustrated that OA significantly counteracted cerebral ischemia-mediated injury through antioxidant effects induced by the regulation of the GSK-3β/HO-1 signaling pathway, implicating OA as a promising neuroprotective drug for the therapy of ischemic stroke.

## 1. Introduction

Ischemic stroke accounts for ~80% of all cases of stroke, which ranks as the second leading cause of death globally [1]. Despite the significant pharmaceutical advances that have been made in recent years, clinically effective drugs for the treatment of ischemic strokes are still lacking [2].

Traditional Chinese medicine (TCM) is considered a huge source of novel drugs and compounds for therapy in neurological diseases [3]. Ginseng (*Panax ginseng*), a popular herb used in TCM, has been confirmed to play a protective role in cerebral ischemia in vivo [4]. Among the bioactive ingredients of ginseng, the triterpenoid oleanolic acid (OA) has exhibited favorable pharmacological properties—including neuroprotective, anticancer, and anti-inflammatory activities [5].

The accumulation of reactive oxygen species (ROS) after an ischemic stroke leads to oxidative stress in the brain, which is one of the fundamental mechanisms underlying neuronal damage caused by ischemic stroke. Hence, antioxidative stress is a potential target in the ischemic stroke therapy [6]. It was reported that OA alleviated cerebral ischemic damage via the modulation of endogenous antioxidants [7], and ameliorated inflammation and apoptosis in PC12 cells induced by oxygen-glucose deprivation/reperfusion (OGD/R) [8]. Thus, OA has been proffered as an effective neuroprotective compound for the treatment of cerebral ischemia via antioxidation [9]; however, a detailed mechanistic understanding of OA’s antioxidation effect on ischemia stroke treatment is still lacking.

Numerous pathways are associated with the regulation of oxidative stress. Heme oxygenase-1 (HO-1) has been reported as the most effective antioxidant-response element (ARE) in the human body, indicating that HO-1 might be a promising therapeutic target in ischemic stroke. Edaravone, marketed in Japan for ischemic stroke treatment, is a free-radical scavenger that functions through the HO-1 pathway [10]. Additionally, glycogen synthase kinase-3β (GSK-3β) is a crucial regulator of HO-1 in controlling oxidative stress [11]. Accumulating evidence has demonstrated that suppression of GSK-3β activity results in overexpression of the HO-1 protein, subsequently ameliorating ischemic stroke-mediated neuronal injury [12,13]. OA was found to improve synaptic connection and neurodegeneration in a mouse model of cerebral ischemia via upregulation of HO-1 [14]. Interestingly, a previous study also demonstrated that pre-treatment with OA protect hepatic ischemia-reperfusion injury through inhibition of GSK-3β [15]. These reports suggest that OA might exert antioxidative effects in ischemic stroke by regulating the GSK-3β/HO-1 pathway, but the supporting evidence is still needed.

Therefore, this study was performed to examine the therapeutic benefits and potential mechanism of OA-mediated amelioration of ischemic brain injury both in vitro and in vivo. OA administration was found to protect neuronal cells against OGD/R damage, as well as alleviate ischemia injury by attenuating oxidative stress in a rat model of middle cerebral artery occlusion (MCAO). These effects might result from regulation of the GSK-3β/HO-1 pathway. The present findings not only provide a novel understanding of the anti-ischemic effects of OA, but also reveal a potential application of OA in treating ischemic stroke.

## 2. Results

### 2.1. OA-Mediated Suppression of OGD/R-Induced Toxicity in SH-SY5Y Cells

**Figure 1:**
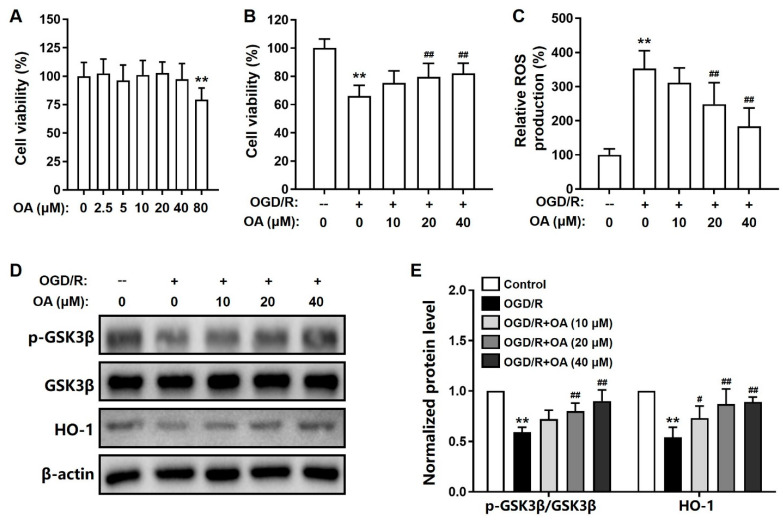
OA suppressed OGD/R-induced toxicity in SH-SY5Y cells via GSK-3β/HO-1 pathway. (**A**) Cell viability of OA on SH-SY5Y cells. (**B**) Cell viability of OA and (**C**) relative ROS production on OGD/R-exposed SH-SY5Y cells with or without OA treatment. (**D**) Representative protein bands and (**E**) quantitative analysis of the p-GSK-3β(Ser9)/GSK-3β ratio and HO-1 protein expression levels in OGD/R-exposed SH-SY5Y cells with or without OA treatment. Data are shown as the mean ± standard deviation (S.D.). ** *p* < 0.01 versus the control group; ^#^ *p* < 0.05 and ^##^ *p* < 0.01 versus the OGD/R group.

We monitored the neuroprotective effects of OA in OGD/R-induced SH-SY5Y cell model of ischemic injury. The cytotoxicity of OA on SH-SY5Y cells was first analyzed, and the cell viability was significantly decreased after OA induction at 80 μM (Figure 1A). Therefore, the dosages of OA used in the in vitro pharmacological study were 10, 20, and 40 μM. As expected, OGD/R induction significantly decreased the viability of SH-SY5Y cells. However, pretreatment with OA significantly suppressed this effect in a dose-dependent manner (Figure 1B). Next, OGD/R-induced ROS production was monitored in SH-SY5Y cells. ODG/R treatment significantly upregulated ROS accumulation than the no-treatment group. OA pretreatment significantly suppressed the elevated ROS production in SH-SY5Y cells in a dose-dependent manner (Figure 1C).

### 2.2. OA Regulates GSK-3β/HO-1 Pathway in OGD/R-Induced SH-SY5Y Cells

Furthermore, the GSK-3β/HO-1 signaling pathway was analyzed using Western blot assays. OGD/R treatment dramatically decreased the ratio of p-GSK-3β(Ser9)/GSK-3β and the expression level of HO-1 in SH-SY5Y cells. As expected, pretreatment with OA significantly ameliorated these effects dose-dependently (Figure 1D,E). These results suggested that pretreatment with OA can suppress OGD/R-induced SH-SY5Y cell injury by regulating the GSK-3β/HO-1 signaling pathway.

### 2.3. OA Administration Attenuated Neurological Deficits and Cerebral Infarction in MCAO Rats

**Figure 2:**
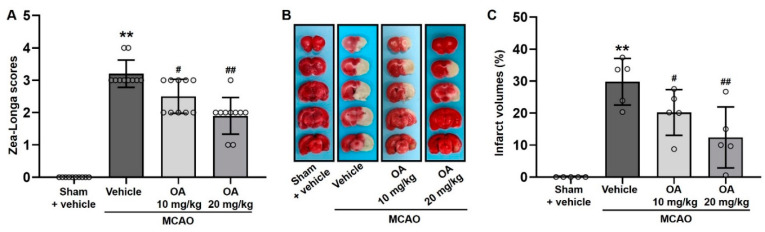
OA administration attenuated neurological deficits and cerebral infarction in MCAO rats. (**A**) Quantitative analysis of neurological function monitored using Zea-Longa scoring. (**B**) Representative images and (**C**) quantitative analysis of infarct volume in the brain (pale area) monitored by TTC staining. Data are shown as the mean ± S.D. ** *p* < 0.01 versus the sham + vehicle group; ^#^ *p* < 0.05 and ^##^ *p* < 0.01 versus the MCAO + vehicle group.

The protective effects of OA on MCAO rats are presented as reductions in neurological deficits and total infarcted area. As shown in Figure 2A, the Zea-Longa scores of the MCAO group rats were significantly increased compared with those of the rats in the sham group, indicating significant MCAO-induced impairment of neurological function. As expected, compared with the MCAO group, OA administration significantly reduced the Zea-Longa score in a dose-dependent manner. In parallel, the volumes of infarcted areas were monitored by TTC staining. As shown in Figure 2B, the MCAO group showed extensive infarcted tissue (pale area) at 6 days post reperfusion. However, no infarcted area was seen in the brains of rats in the sham group (Figure 2B). Quantitative analysis was conducted for comparing the infarct volume (Figure 2C). The infarct volume of the brains in the MCAO group were dramatically increased compared to those in the sham group which had no infarct volume. As respected, OA-treated MCAO rats had significantly and dose-dependently reduced infarct volumes compared to untreated MCAO rats (Figure 2C). These results indicated that OA significantly ameliorated ischemic brain injury in rats with MCAO-induced cerebral ischemia.

### 2.4. OA Administration Reduced Neuronal Damage in MCAO Rats

**Figure 3:**
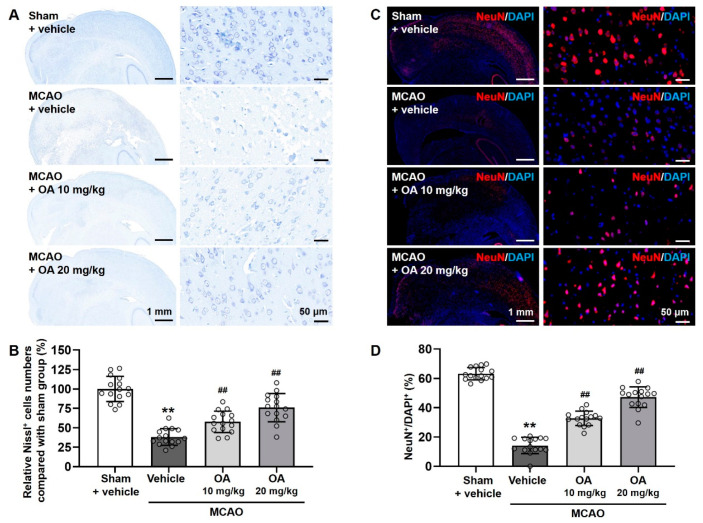
OA administration reduced neuronal damage in MCAO rats. (**A**) Representative images of Nissl staining and (**B**) quantitative analysis of the proportion of Nissl^+^ cells in the infarcted area in MCAO rats with or without OA administration. (**C**) Representative images of NeuN immunofluorescent staining and (**D**) quantitative analysis of the proportion of NeuN^+^ cells in the infarcted area in MCAO rats with or without OA administration. Data are shown as the mean ± S.D. Scale bars: 1 mm and 50 μm. ** *p* < 0.01 versus the sham + vehicle group; ^##^ *p* < 0.01 versus the MCAO + vehicle group.

Neuronal damage in MCAO rats was monitored by Nissl staining and immunofluorescent staining of NeuN in the infarcted areas. Nissl staining revealed a significant reduction in the proportion of Nissl^+^ cells in the infarcted areas of the rats in the MCAO group compared to the sham group, indicating neuronal degradation in the former. However, the proportion of Nissl^+^ cells was significantly upregulated in OA-treated MCAO rats compared to untreated MCAO rats in a dose-dependent manner (Figure 3A,B). Consistently, immunofluorescent NeuN staining revealed that the proportion of NeuN^+^ cells in the infarct areas was dramatically decreased in the MCAO group compared to the sham group. This reduction was markedly ameliorated following OA administration in a dose-dependent manner (Figure 3C,D). These findings suggested that OA administration significantly and dose-dependently ameliorated neuronal damage in infarcted regions in MCAO rats.

### 2.5. OA Administration Reduced Cellular Apoptosis in MCAO Rats

**Figure 4:**
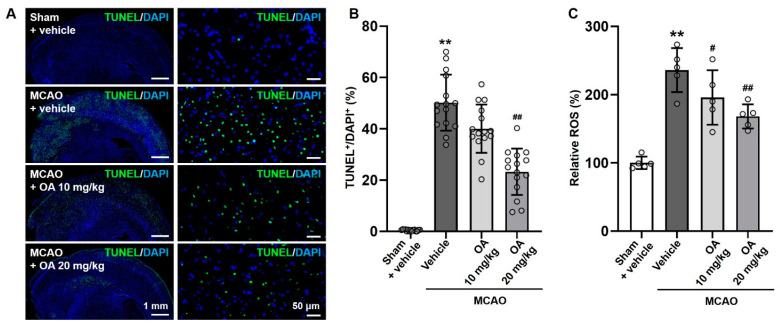
OA administration reduced cellular apoptosis in MCAO rats. (**A**) Representative images of TUNEL staining and (**B**) quantitative analysis of the proportion of TUNEL^+^ cells in the infarcted area in MCAO rats with or without OA administration. (**C**) Relative ROS production in MCAO rats with or without OA administration. Data are shown as the mean ± S.D. Scale bars: 1 mm and 50 μm. ** *p* < 0.01 versus the sham + vehicle group; ^#^ *p* < 0.05 and ^##^ *p* < 0.01 versus the MCAO + vehicle group.

TUNEL staining was performed to monitor cellular apoptosis in the MCAO rats. The proportion of TUNEL^+^ cells in the infarcted regions was significantly upregulated in the MCAO group compared to the sham group. As expected, this increase was abolished in OA-treated MCAO rats compared to untreated MCAO rats in a dose-dependent manner. In particular, compared with the untreated MCAO group, the percentage of TUNEL^+^ cells in cerebral infarct tissue significantly decreased in MCAO rats after treatment with 20 mg/kg OA (Figure 4A,B).

Furthermore, the MCAO group showed significantly upregulated ROS levels in cerebral infarct tissue compared to the sham group. As expected, OA administration significantly and dose-dependently prevented this increase in ROS production (Figure 4C). The results strongly indicated that OA administration could inhibit MCAO-induced neuron apoptosis and oxidative stress in infarcted regions.

### 2.6. OA Administration Regulated GSK-3β/HO-1 Signaling Pathway

**Figure 5:**
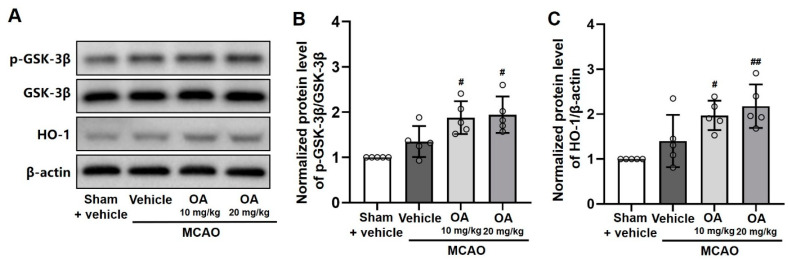
OA administration regulated GSK-3β/HO-1 signaling pathway. (**A**) Representative protein bands and quantitative analysis of the (**B**) p-GSK-3β(Ser9)/GSK-3β ratio and (**C**) HO-1 protein expression levels, as assessed by Western blots, in the infarcted tissue of MCAO rats with or without OA administration. Data are shown as the mean ± S.D. ^#^ *p* < 0.05 and ^##^ *p* < 0.01 versus the MCAO + vehicle group.

The role of the GSK-3β/HO-1 pathway in OA-mediated neuroprotection in MCAO rats was examined using Western blot assays. The ratio of p-GSK-3β(Ser9)/GSK-3β and the HO-1 protein expression levels were not significantly changed in the infarcted tissue of MCAO rats compared to sham rats (Figure 5A–C). However, compared with untreated MCAO rats, OA-treated rats (both 10 and 20 mg/kg OA) showed a significantly increased p-GSK-3β(Ser9)/GSK-3β ratio (Figure 5B), as well as an increase in the expression of HO-1 protein (Figure 5C), in a dose-dependent manner. These results indicated that GSK-3β/HO-1 signaling was crucial for neuroprotection in MCAO rats following OA administration.

## 3. Discussion

The burden of stroke worldwide is expected to increase further as a result of the increasing aging population [16]. To date, pharmacological interventions to promote stroke rehabilitation have been studied in clinical and preclinical settings. However, most of these interventions have failed due to the ambiguous efficacy and safety in humans with stroke [17]. Therefore, it is crucial to identify novel neuroprotective agents to both prevent and treat ischemic stroke.

Multifarious therapeutic strategies have been developed for ischemic stroke treatment. Thrombolysis is one of the most effective treatments, but it has been shown to increase the risk of symptomatic intracranial hemorrhage [18]. Recently, cellular therapies—including induced pluripotent stem cells or neural stem cells—have been shown to have the potential to contribute neuronal cells’ viability following ischemic injury. However, such therapies are still under development and may increase the risk of tumorigenesis [19]. Pharmacotherapy is preferred for the patients with ischemic stroke. Considering the central role of oxidative stress in stroke pathogenesis, antioxidative agents—especially natural compounds—have been considered to be a potentially effective therapeutic strategy for ischemic stroke [20].

Ginseng (*Panax ginseng*) and its components are known to possess significant antioxidant effects and may help prevent and treat several diseases—including cancer, cardiovascular, and nervous system disorders [21]. OA, a natural pentacyclic triterpenoid, is a bioactive ingredient of ginseng that can penetrate the blood–brain barrier [22]. Several studies have demonstrated that OA significantly ameliorated cognitive declines in the mouse model of Alzheimer’s disease at 10 mg/kg and in the rat model of Alzheimer’s disease at 21.6 mg/kg [23,24]. Earlier research also reported that OA improved depressant-like behaviors in mice at the dosage of 10 and 20 mg/kg [25]. Noteworthy, it was reported that the liver injury was observed and the bodyweight was significantly lost in adult C57BL/6 mice after OA administration at 90 mg/kg or above for 5 days [26]. These studies suggested that OA produced significantly neuropharmacological properties at around 10–20 mg/kg and might exhibit potentially toxic effects at a higher dosage. In the present study, the dose selection of OA, 10 and 20 mg/kg, was based on selecting the optimal dose of OA that balances the effects and risks. The results of our study demonstrated that OA significantly reduced the neurological deficit of MCAO rats at 10 and 20 mg/kg.

Accumulating evidence has revealed that the GSK-3β/HO-1 pathway modulates oxidative stress levels in the progression and treatment of ischemic stroke [27,28]. An earlier study suggested that oleanolic triterpenoid affected cell migration via inhibition of GSK-3β activity [29], and an in silico study also hypothesized that OA may exert wound-healing activity by inhibiting GSK-3β [30]. Consistently, the present study showed that OA administration can significantly inhibit GSK-3β activation and consequently increase HO-1 expression, resulting in a reduction in the pathological alterations induced in the MCAO rat model, as well as protect neuronal cells against OGD/R-induced damage. However, it is worth mentioning that GSK-3β is essential for brain development, neuronal plasticity, and other normal human functions [31]. The safety and feasibility of using GSK-3β regulators, such as OA, for treating ischemic stroke should be carefully monitored in the future.

Several recent studies investigated the antioxidant activity of OA in different disease models. For example, OA was found to reduce oxidative stress in silicotic rats by modulating the Akt/NF-κB pathway [32]. Moreover, OA suppressed oxidative stress by regulating the stanniocalcin-1 pathway in a cell model of Alzheimer’s disease treatment [33], as well as repressed oxidative stress via the SIRT3/NF-κB axis in an in vitro osteoarthritis cell model [34]. These studies indicate that such OA-mediated antioxidant effects are broadly applicable, and that the underlying mechanisms are complex. As such, the involvement of other mechanisms, in addition to the GSK-3β/HO-1 pathway-mediated effects, during OA-mediated treatment of ischemic stroke is unclear and warrants further investigation. As the population ages, neurodegenerative diseases—including stroke—have been identified as one of the greatest public health problems. Although there are currently no effective treatments for neurodegenerative diseases, antioxidants are considered as a promising approach to slow the progression of and treat these disorders [35]. The present study offers insight into the development of natural compounds, such as OA, as novel treatments of neurodegeneration diseases.

## 4. Materials and Methods

### 4.1. Cell Culture and the OGD/R Model

SH-SY5Y cells, purchased from ATCC, have been extensively used in studies of cerebral ischemia. SH-SY5Y cells were cultured in DMEM supplemented with 10% fetal bovine serum (FBS) and 1% penicillin/streptomycin at 37 °C in a 5% CO_2_ humidified incubator. Cells were incubated with different OA concentrations (10, 20, and 40 μM) for 12 h prior to induction of OGD/R and then cultured in glucose-free medium without FBS under hypoxic conditions as described above for 4 h. Then, the cells were incubated in a normoxic incubator with normal culture medium for reoxygenation.

### 4.2. MTT Assay

OA (≥98%, HPLC grade) was purchased from Must Bio-Technology Co., Ltd. (Chengdu, China). The MTT assay was carried out for a cell viability test. After treatment with OA and OGD/R, MTT solution (5 mg/mL) was added and incubated for another 4 h. Then, the solution was replaced by dimethyl sulfoxide (DMSO) to dissolve the formazan crystals. Absorbance was monitored at 570/630 nm (excitation/emission) with a microplate reader. Relative cell viability was expressed as the absorbance of each well content relative to the corresponding untreated well content.

### 4.3. Measurement of ROS in Cells

Intracellular ROS production was analyzed by a fluorescent (5,6)-chloromethyl-2′,7′-dichlorodihydrofluorescein diacetate, acetyl ester (CM-H_2_DCFDA) probe (Invitrogen, Carlsbad, CA, USA) according to the manufacturer’s instructions. The fluorescence intensity was measured using a microplate reader at 495/525 nm (excitation/emission).

### 4.4. Western Blot Assay

Proteins were extracted from the infarcted tissue and cultured cells using RIPA buffer. The proteins were separated by SDS-PAGE and transferred to PVDF membranes using the Bio-Rad transfer system (Bio-Rad Laboratories, Hercules, CA, USA). The membranes were blocked with 5% fat-free milk and incubated with corresponding primary antibodies at 4 °C overnight, including GSK-3β antibody (Cat.: 9315S, Cell Signaling Technology, Danvers, MA, USA), p-GSK-3β (Ser9) antibody (Cat.: 5558S, Cell Signaling Technology), HO-1 antibody (Cat.: 86806S, Cell Signaling Technology), and β-actin antibody (Cat.: A5316, Sigma-Aldrich, St. Louis, MO, USA). After washing, the membranes were incubated with secondary antibodies. The blots were visualized using a chemiluminescence kit (Millipore, Burlington, CA, USA) and evaluated using the ChemiDoc Touch imaging system (Bio-Rad Laboratories). Relative band intensities were quantified using ImageJ (NIH, Bethesda, MD, USA).

### 4.5. Animals and OA Administration

Healthy male Sprague-Dawley rats (200–220 g weight) were purchased from Viton Lihua Experimental Animal Technology Co., Ltd. (Beijing, China). All rats were housed in a 12 h light/dark cycle and humidity- and temperature-controlled environment with ad libitum access to food and water.

Experimental protocols were approved by the Department of Health, the Government of Hong Kong Special Administrative Region. The rats were randomly grouped as follows (*n* = 10 per group): sham + vehicle, MCAO + vehicle, MCAO + OA (10 mg/kg), and MCAO + OA (20 mg/kg). OA was prepared in saline solution with 2% Tween-80. Drug administration was carried out 3 days pre-MCAO and 6 days post-MCAO via intraperitoneal injection once daily. Rats in sham and untreated MCAO groups were given the equivalent volume of vehicle. The OA doses were selected according to previous reports [23,24,25,26].

### 4.6. MCAO Procedure

MCAO was induced after the third administration of OA as described in our previous report [36]. Briefly, rats were anesthetized by 3% isoflurane inhalation (1.5 L/min). The arterial sheath was carefully separated without injuring the vagus nerve, followed by separation of the common carotid artery (CCA), external carotid artery (ECA), and internal carotid artery (ICA) with a midline incision. The CCA and ECA were ligated, and a silicon-coated monofilament suture was inserted into the ECA and advanced through the ICA to block blood flow and occlude the middle cerebral artery (MCA). The monofilament was withdrawn to restore blood circulation after 1.5 h occlusion and allows reperfusion. Sham rats were subjected to the same surgical processes but without MCAO.

### 4.7. Neurological Deficit Assessment and Brain Tissue Collection

Neurological function was analyzed 6 days after reperfusion using the Zea-Longa score, as described previously [36]. The Zea-Longa score ranges from 0 to 4 (0: no obvious impairment; 1: inextensibility of the contralateral forepaw; 2: circling to the contralateral side; 3: leaning to the contralateral side; 4: disability to walk spontaneously).

After assessing neurological status, all rats were perfused with phosphate-buffered saline under anesthesia prior to the collection of brain tissues. Five whole brains were collected from each group for TTC staining. Another five brains were divided into anterior and posterior hemispheres. The anterior hemispheres were used for histopathological staining, while the posterior hemispheres were stored at −80 °C and used for ROS measurement and Western blot assays.

### 4.8. TTC Staining

The extent of the infarcts was monitored by TTC staining. The whole brains (*n* = 5) were sliced into coronal sections and stained with a 2% TTC (Sigma-Aldrich) solution at 37 °C for 20 min, followed by fixation with 10% formaldehyde. The volumes of infarcted areas (pale) and non-infarcted areas (red/pink color) were quantified by using Image J software. The infarcted volume was calculated as the percentage of the infarcted area relative to the total hemisphere area.

### 4.9. Nissl and Immunofluorescent Staining

Neuronal loss in the infarcted area was assessed by Nissl and immunofluorescent NeuN staining. Brain hemispheres (*n* = 5) were fixed in 10% formaldehyde solution, embedded in paraffin, and micro-sectioned into coronal sections. The sections were stained using Nissl staining solution (Beyotime, Beijing, China) as per the manufacturer’s instructions. For immunofluorescent NeuN staining, coronal brain sections were incubated with an anti-NeuN antibody at 4 °C overnight, followed by incubation with FITC-conjugated secondary antibody. DAPI was used to stain the cell nuclei. Three images in the infarct area were randomly selected from each brain. The relative Nissl^+^ cell numbers and proportion of NeuN^+^/DAPI^+^ cells in the infarcted region were calculated. All images were captured using a Pannoramic DESK scanner and analyzed using the CaseViewer software (3DHISTECH, Budapest, Hungary).

### 4.10. TUNEL Staining

Neuronal cell apoptosis was monitored by TUNEL staining using an in-situ Cell Death Detecting kit (Roche Diagnostics GmbH, Mannheim, Germany), as previously described [13]. Cell nuclei were counterstained with DAPI. Three images in the infarct area were randomly selected from each brain. The proportion of TUNEL^+^/DAPI^+^ cells in the infarcted region was calculated. All images were captured using a Pannoramic DESK scanner and analyzed using the CaseViewer software.

### 4.11. ROS Quantification in Tissue

Infarcted tissue homogenate (*n* = 5) was centrifuged, and the supernatant was used to quantify ROS using a ROS assay kit (Nanjing Jiancheng Bioengineering Institute, Nanjing, China) as per the manufacturer’s instructions. ROS production was monitored using a microplate reader (Perkin Elmer, Eden Prairie, MN, USA), and relative ROS levels were calculated.

### 4.12. Statistical Analyses

The data were presented as the means ± S.D. Statistical analyses were assessed with one-way ANOVA using SPSS (Version 24.0). The statistical significance level was set at *p* < 0.05.

## 5. Conclusions

In conclusion, this study demonstrated that OA administration can prevent stroke-associated pathological changes by inducing antioxidative effects via regulating the GSK-3β/HO-1 pathway both in vitro and in vivo. Beyond ischemic stroke, natural antioxidative compounds that regulate the GSK-3β/HO-1 signaling pathway may hold significant potential in the treatment of aging-related neurodegenerative diseases.

## Data Availability

Data is contained within the article.
